# Resting-State Brain Network Dysfunctions Associated With Visuomotor Impairments in Autism Spectrum Disorder

**DOI:** 10.3389/fnint.2019.00017

**Published:** 2019-05-31

**Authors:** Zheng Wang, Yan Wang, John A. Sweeney, Qiyong Gong, Su Lui, Matthew W. Mosconi

**Affiliations:** ^1^Department of Occupational Therapy, University of Florida, Gainesville, FL, United States; ^2^Huaxi Magnetic Resonance Research Center, Department of Radiology, West China Hospital of Sichuan University, Chengdu, China; ^3^Department of Psychiatry and Behavioral Neuroscience, University of Cincinnati College of Medicine, Cincinnati, OH, United States; ^4^Schiefelbusch Institute for Life Span Studies, University of Kansas, Lawrence, KS, United States; ^5^Clinical Child Psychology Program, University of Kansas, Lawrence, KS, United States; ^6^Kansas Center for Autism Research and Training, University of Kansas, Lawrence, KS, United States

**Keywords:** autism spectrum disorder, resting-state functional MRI, visuomotor control, precision grip, cortical–cerebellar connectivity, amplitude of low-frequency fluctuations, functional connectivity

## Abstract

**Background:** Individuals with autism spectrum disorder (ASD) show elevated levels of motor variability that are associated with clinical outcomes. Cortical–cerebellar networks involved in visuomotor control have been implicated in postmortem and anatomical imaging studies of ASD. However, the extent to which these networks show intrinsic functional alterations in patients, and the relationship between intrinsic functional properties of cortical–cerebellar networks and visuomotor impairments in ASD have not yet been clarified.

**Methods:** We examined the amplitude of low-frequency fluctuation (ALFF) of cortical and cerebellar brain regions during resting-state functional MRI (rs-fMRI) in 23 individuals with ASD and 16 typically developing (TD) controls. Regions of interest (ROIs) with ALFF values significantly associated with motor variability were identified for for patients and controls respectively, and their functional connectivity (FC) to each other and to the rest of the brain was examined.

**Results:** For TD controls, greater ALFF in bilateral cerebellar crus I, left superior temporal gyrus, left inferior frontal gyrus, right supramarginal gyrus, and left angular gyrus each were associated with greater visuomotor variability. Greater ALFF in cerebellar lobule VIII was associated with less visuomotor variability. For individuals with ASD, greater ALFF in right calcarine cortex, right middle temporal gyrus (including MT/V5), left Heschl's gyrus, left post-central gyrus, right pre-central gyrus, and left precuneus was related to greater visuomotor variability. Greater ALFF in cerebellar vermis VI was associated with less visuomotor variability. Individuals with ASD and TD controls did not show differences in ALFF for any of these ROIs. Individuals with ASD showed greater posterior cerebellar connectivity with occipital and parietal cortices relative to TD controls, and reduced FC within cerebellum and between lateral cerebellum and pre-frontal and other regions of association cortex.

**Conclusion:** Together, these findings suggest that increased resting oscillations within visuomotor networks in ASD are associated with more severe deficits in controlling variability during precision visuomotor behavior. Differences between individuals with ASD and TD controls in the topography of networks showing relationships to visuomotor behavior suggest atypical patterns of cerebellar–cortical specialization and connectivity in ASD that underlies previously documented visuomotor deficits.

## Introduction

Autism spectrum disorder (ASD) is characterized by difficulties in social interaction and communication, a restricted repertoire of interests and stereotypic behaviors (American Psychiatric Association, 2013). Sensorimotor deficits are common in ASD including reduced accuracy of ballistic and smooth pursuit eye movements (Takarae et al., 2007; Schmitt et al., 2014), gait abnormalities (Esposito and Ventola, 2008; Calhoun et al., 2011), macrographia (Fuentes et al., 2009), and atypical finger mannerisms (Anzulewicz et al., 2016). Reduced sensorimotor control interferes with multiple adaptive skills (Travers et al., 2016), and more severe motor deficits appear to be related to more severe social (Haswell et al., 2009; Landa et al., 2016), cognitive (Estes et al., 2015), and language impairments in ASD (Iverson, 2010).

Our group has documented that individuals with ASD show increased force variability relative to typically developing (TD) controls during visually guided precision gripping (Mosconi et al., 2015; Wang et al., 2015) and isometric index finger abduction (Wang et al., 2017). Precision force control is essential for manual dexterity, and increased grip force variability is associated with diminished capacity to execute manual motor tasks such as writing, buttoning clothes, and manipulating small or delicate items (Potter et al., 2009). Non-human primate and task-based functional magnetic resonance imaging (tb-fMRI) studies have delineated distinct cortical–cerebellar networks involved in reactively adjusting sustained precision motor behavior in response to visual information (Kelly and Strick, 2003; Vaillancourt et al., 2006; Coombes et al., 2010). Specifically, visual–spatial information is processed in calcarine cortex and relayed to posterior parietal cortex (Glickstein, 2000). Visual feedback information is translated directly to ventral and dorsal premotor cortex, and then to primary motor cortex in order to adjust outgoing motor commands. Additionally, parietal–ponto–cerebellar–thalamo–motor cortical pathways (Glickstein, 2000; D'Mello and Stoodley, 2015) integrate sensory feedback error information to refine motor outputs at the periphery. Within the cerebellum, anterior (I–V) and inferior (VIII–X) lobules are densely connected with somatomotor and brainstem circuits and are involved in basic sensorimotor behaviors (Nitschke et al., 2005; Stoodley and Schmahmann, 2009, 2010). In contrast, the more phylogenetically advanced lateral hemispheres (crus I–II) innervate pre-frontal and association cortices via dentate nuclei and thalamus (Ramnani, 2006). The extent to which these distinct cortical–cerebellar circuits are functionally affected and associated with visuomotor impairments in ASD is not yet known.

Consistent and growing evidence from neuroimaging studies suggests that ASD is characterized by abnormalities of distributed functional networks, rather than focal impairment. Task-based fMRI (tb-fMRI) studies have documented reduced activation of cortical–cerebellar networks accompanied by increased recruitment of supplementary motor area during simple sequential finger tapping (Mostofsky et al., 2009). Using diffusion tensor imaging, several studies have demonstrated reduced white matter microstructural integrity within fronto–parietal networks (Fitzgerald et al., 2018), cortical–basal ganglia networks (Barnea-Goraly et al., 2004; Shukla et al., 2010; Nair et al., 2015), brainstem (Hanaie et al., 2016), and both middle and superior peduncles of the cerebellum in ASD (Catani et al., 2008; Brito et al., 2009; Hanaie et al., 2013). These studies suggest that aberrant functional and structural connectivity of cortical and subcortical networks supporting sensorimotor control may contribute to sensorimotor impairments in ASD.

The examination of brain activity during resting-state fMRI (rs-fMRI) is a well-validated approach that allows characterization of intrinsic properties of regional- and network-level functional activation and connectivity (Biswal et al., 1995; Fox and Raichle, 2007). During rest, the brain displays spontaneous low-frequency (0.01–0.08 Hz) blood oxygen level-dependent (BOLD) fluctuations reflecting neural activity when goal-directed cognitive behavioral actions and external sensory inputs are minimized relative to active task conditions (Biswal et al., 1995; Zuo et al., 2010). Importantly, these low-frequency fluctuations during rest show high levels of temporal correlation with discrete proximal and distal brain regions that comprise specialized brain networks involved in cognition and behavior as determined by tract-tracing, histopathological, and tb-fMRI studies (Fox and Raichle, 2007; Fox et al., 2007; Ma et al., 2011). Moreover, coherent intrinsic BOLD fluctuations account for a majority of the BOLD–behavior relationship observed during tb-fMRI (Fox et al., 2006, 2007). For example, the resting amplitude of low-frequency fluctuations (ALFFs) and functional connectivity (FC) of frontal–parietal motor networks are strongly associated with the rates at which individuals are able to learn novel manual motor skills (Ma et al., 2011).

Few studies have characterized regional ALFF during rest in ASD, although several rs-fMRI studies have documented atypical FC in patients relative to controls (Just et al., 2004; Jones et al., 2010; Tyszka et al., 2014; Cerliani et al., 2015; Hull et al., 2017). A consistent observation of these studies includes reduced long-distance cortical FC in individuals with ASD, with effects most pronounced within sensorimotor, default mode, and visual perceptual networks (Assaf et al., 2010; Di Martino et al., 2014; Hahamy et al., 2015). Recent studies showed decreased cerebellar connectivity to somatomotor and visual cortices in ASD relative to controls that was related to more severe ASD symptoms (Khan et al., 2015; Cardon et al., 2017). These findings suggest that intrinsic functional communication between discrete regions of visual–motor brain networks may be selectively impaired in ASD and related to key clinical features of the disorder. It remains unclear whether intrinsic ALFF and FC of these networks are associated with visuomotor impairments in ASD.

The present study aimed to characterize intrinsic functional properties of distinct brain regions and networks associated with visually guided precision motor control in individuals with ASD and matched TD controls. ALFF and FC were quantified during rs-fMRI and compared with precision grip force variability during a visuomotor task previously studied in ASD (Mosconi et al., 2015; Wang et al., 2015). Based on prior findings showing increased involvement of “non-visuomotor” networks in ASD during simple motor tasks (Takarae et al., 2007; Mostofsky et al., 2009), we used a data-driven rather than a pre-defined region of interest (ROI) approach to identify cortical and subcortical brain regions associated with force variability. This approach has the advantage of identifying relevant ROIs that are outside primary sensorimotor networks but still associated with visuomotor behavior. FC between brain regions identified as significant in our ALFF–force variability analysis was then compared between groups and examined in relation to visuomotor behavior. Given prior findings that more severe motor abnormalities (Haswell et al., 2009; Estes et al., 2015; Landa et al., 2016) and FC alterations (Khan et al., 2015; Cardon et al., 2017) of visuomotor networks are associated with more severe ASD symptoms, we also examined the relationship between visuomotor network ALFF and FC with clinical ratings of social–communication abnormalities and restricted and repetitive behaviors in individuals with ASD.

## Methods

### Participants

Twenty-three participants with ASD and 16 healthy controls completed a rs-fMRI scan. Of these participants, 10 individuals with ASD and 11 age-, gender-, and IQ-matched controls also completed a task of visually guided precision gripping during a separate tb-fMRI run (Table 1). Among the participants who completed the rs-fMRI run, 12 (ASD = 10, controls = 2) were not administered the tb-fMRI procedure, as the protocol was developed subsequent to the rs-fMRI study initiation. Six participants (ASD = 3, controls = 3) completed the rs- and tb-fMRI studies, but their data were not included due to technical or task compliance issues (e.g., intermittent relaxation of force during the task).

**Table 1:** Demographic and diagnostic characteristics [mean (SD)] for TD controls and individuals with ASD.

	**Individuals who completed both tb-fMRI and rs-fMRI**			**Individuals who completed rs-fMRI**		
	**Controls (*n* = 11)**	**ASD (*n* = 10)**	***p***	**Controls (*n* = 16)**	**ASD (*n* = 23)**	***p***
Age in years	22.82 (4.38)	21 (5.58)	0.414	23.31 (4.11)	19.09 (5.90)	0.018^†^
*Range*	*17–33 years*	*14–32 years*		*17–33 years*	*10–32 years*	
% Male^a^	90.9%	100%	1.000	93.8%	91.3%	1.000
FSIQ	120.70 (11.19)	109.90 (16.05)	0.098	121.07 (11.25)	101.78 (16.43)	0.000^***^
*Range*	*94–133*	*79–129*		*94–138*	*78–129*	
PIQ	118.80 (11.60)	108.80 (15.85)	0.125	118.40 (10.44)	104.00 (14.87)	0.002^**^
*Range*	*94–133*	*83–129*		*94–133*	*79–129*	
VIQ	118.00 (12.03)	109.10 (16.27)	0.181	118.87 (12.55)	99.39 (18.50)	0.001^**^
*Range*	*95–135*	*80–133*		*95–140*	*64–133*	
ADOS social		9.70 (3.89)			9.68 (3.68)	
*Range*		*4–16*			*4–16*	
ADOS RRB		2.70 (2.00)			2.68 (1.55)	
*Range*		*0–7*			*0–7*	
RBS-R total		23.14 (13.68)			26.85 (19.74)	
*Range*		*9–47*			*0–78*	

*FSIQ, full scale IQ; PIQ, performance IQ; VIQ, verbal IQ; ADOS Social, ADOS social affect algorithm score; ADOS RRB, ADOS restricted and repetitive behaviors algorithm score; RBS-R total, repetitive behaviors scale-revised total score*.

a *Chi-square (χ^2^) statistics using Fisher's exact test*.

† p < 0.05,

* p < 0.01,

** p < 0.005,

*** *p < 0.001*.

Individuals with ASD were recruited through community advertisements and local clinical programs. All participants with ASD met classification criteria for autism on the Autism Diagnostic Inventory-Revised (ADI-R) (Lord et al., 1994) and for autism or autism spectrum on the Autism Diagnostic Observation Schedule (ADOS) and were diagnosed with ASD according to DSM-5 criteria based on expert clinical opinion (American Psychiatric Association, 2013). IQ was assessed using the Wechsler Abbreviated Scales of Intelligence (Wechsler, 2011). No individuals with ASD had any known genetic syndrome associated with ASD (e.g., fragile X syndrome).

Control participants were recruited from the community and had a score of eight or lower on the Social Communication Questionnaire (SCQ) (Rutter et al., 2003). Control participants were excluded for current or past psychiatric or neurological disorders, family history of ASD in first- or second-degree relatives, or a history in first-degree relatives of a developmental or learning disorder, psychosis, or obsessive–compulsive disorder. No participants were taking medications known to affect motor performance at the time of testing, including antipsychotics, stimulants, or anticonvulsants (Reilly et al., 2008). No participant had a history of head injury, birth asphyxia, or non-febrile seizure. Study procedures were approved by the local Institutional Review Board. Adult participants provided both informed and written consent, and minors provided assent in addition to written consent from their legal guardian.

### Visuomotor Data Acquisition

MRI scanning was completed on an Achieva 3-Tesla Philips system (Philips Medical Systems, Andover, MA). Each scanning session included a T1-weighted high-resolution structural scan (repetition time = 8.1 ms; echo time = 3.373 ms; flip angle = 12°; field of view = 256 × 204 × 160 mm^3^; matrix = 256 × 204 × 160; 160 sagittal slices; voxel size = 1 mm^3^; no gap). The T1 scan was performed prior to functional scans to facilitate functional data registration to standardized space.

**Figure 1:**
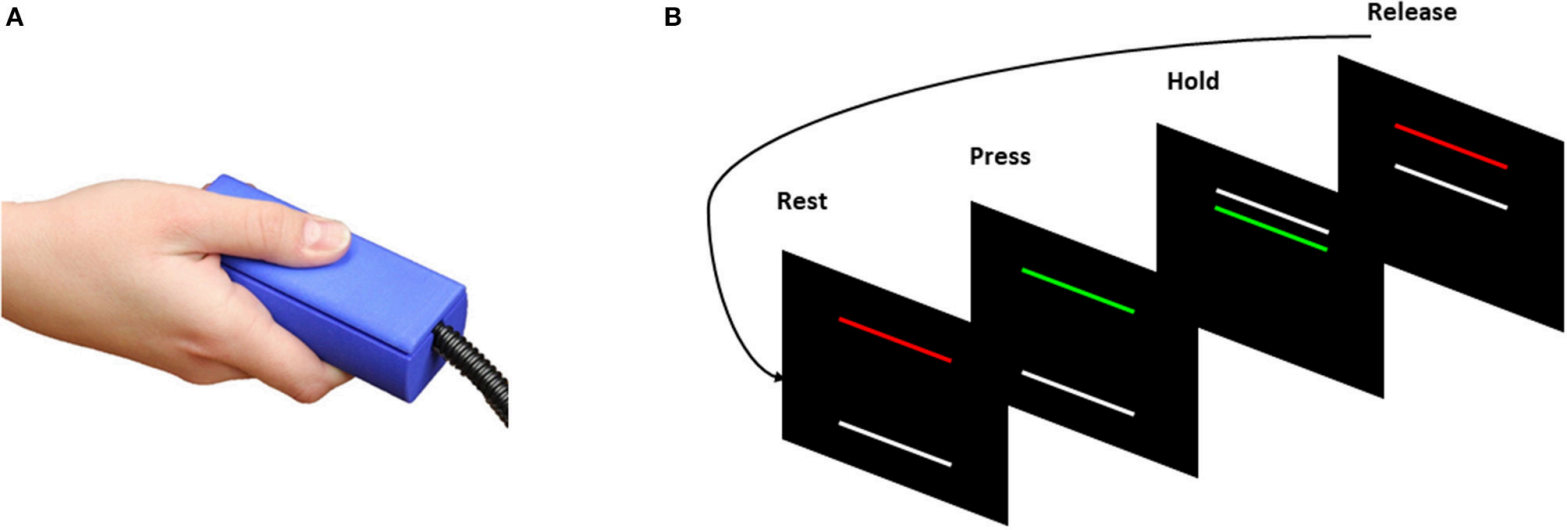
**(A)** Individuals pressed against the Bragg grating fiber optic force transducer during a test of precision grip. **(B)** Visual information individuals received during task-based fMRI (tb-fMRI). Participants viewed two horizontal bars presented against a black background (Rest/Release). The TARGET bar (red/green) was stationary during each trial. The TARGET bar turned from red to green at the beginning of each trial to cue participants to begin pressing the force transducer. The white FORCE bar moved upward with increased force. The discrepancy between the TARGET and FORCE bars provided online visual feedback to the participants about their motor performance.

Experimental procedures for the fMRI visuomotor test were similar to laboratory tests reported previously from our group (Mosconi et al., 2015; Wang et al., 2015). Prior to MR imaging, each participant's maximum voluntary contraction (MVC) was measured using a custom Bragg grating fiber optic force transducer (Model sm130; Neuroimaging Solutions, Gainesville, FL). The transducer was housed in a precision grip apparatus. Participants were instructed to hold the apparatus using the right thumb, middle, and index fingers (Figure 1; e.g., Neely et al., 2016). Participants then were instructed to press as hard as they could for three 5-s trials, and the average of their maximum force during these trials was used as their MVC.

During the scan, participants rested their hands at their sides and used their right hand to grip the force transducer without moving their arm while viewing visual feedback regarding their performance. A stationary TARGET bar (red/green) located in the middle of the screen was set to 60% of each individual's MVC. The TARGET bar turned from red to green at the beginning of each trial to cue participants to begin pressing the gripping device. Participants' motor performance was represented as a white FORCE bar that moved upward with increases in force output. Participants were instructed to press on the transducer so that the white FORCE bar reached and stayed at the same level as the stationary TARGET bar.

The visual angle of the FORCE bar was set to 0.623 as we have done previously (Mosconi et al., 2015). Participants completed five 24-s blocks in which they pressed on the transducer while receiving visual feedback. Each run began and ended with 24-s rest blocks, and each force block was separated by 24-s rest blocks (total scan time: 4:50). Only participants successfully completing at least three force trials were included in final analyses.

### Resting-State fMRI Data Acquisition

During a 5-min rs-fMRI scan (240 brain volumes; 33 interleaved axial slices per volume; TR = 1,500 ms; echo time = 25 ms; flip angle = 60°; field of view = 220 × 114.2 × 220 mm^3^; voxel size = 3.438 × 3.438 × 3.4 mm^3^; 1-mm gap), participants were instructed to keep their eyes closed and refrain from any cognitive, language, or motor behavior as much as possible. Participants were queried regarding their ability to stay awake following each run; only runs in which participants reported staying awake were included in analyses.

Seed ROIs associated with visuomotor behavior were identified using the subset of participants who completed tb-fMRI (10 individuals with ASD and 11 matched controls). Clusters with significant correlations between ALFF and sustained force variability were defined separately for the ASD and TD groups. FC analysis was performed using the significant ROIs identified in these ALFF–force variability analyses as seed regions, but then analyzed across all participants who completed the rs-fMRI study as described below.

### Visuomotor Data Processing and Analysis

Each force trace was low-pass filtered *via* a double-pass fourth-order Butterworth filter at a cutoff of 15 Hz in Matlab 2017a (MathWorks, Inc., Natick, MA). The first and last 3 s of each trial were excluded from the analyses to reduce variability related to the rates at which individuals initially increased their force level or relaxed their force at the end of trials. Thus, the middle 18 s of each sustained force trial was analyzed. To quantify individuals' motor variability, we examined the standard deviation of force during each trial for each individual. Mean force of each trial was also analyzed to ensure that participants understood and completed the task.

### Rs-fMRI Data Processing

The rs-fMRI pre-processing was performed using the Data Processing Assistant for Resting-State fMRI 3.0 toolbox (DPARSF 3.0; http://rfmri.org/DPARSF) in MATLAB. The first 10 volumes of each run were discarded to reduce artifacts caused by magnetic instability (Zhang et al., 2012). Volumes were slice-time corrected and realigned to the middle slice. Rigid body realignment parameters were estimated for each individual, and data were excluded if individuals showed head motion ≥3 mm in the horizontal plane or over 3° in rotation. The rs-fMRI data of one individual with ASD were removed from the original dataset due to excessive head motion. There were no significant differences between individuals with ASD and controls for any of the six motion parameters (three translational and three rotational) (Supplementary Table 1).

Each individual's rs-fMRI data were registered to their own T1-weighted anatomical scan and spatially normalized to Montreal Neurological Institute (MNI) space using the unified segmentation–normalization algorithm in SPM8 (http://www.fil.ion.ucl.ac.uk/spm/software/spm8/). Specifically, individuals' structural images were coregistered to the functional image after motion correction, and the transformed structural images were segmented into gray matter, white matter, and cerebrospinal fluid by using a unified segmentation algorithm. The motion corrected functional images were spatially normalized to MNI space and resampled to 3 × 3 × 3 mm voxels using the normalization parameters estimated during unified segmentation. We regressed out nuisance covariates including linear trends, friston 24 head motion parameters, white matter, and cerebrospinal fluid signal. Data were spatially smoothed using a 4-mm full width at half maximum (FWHM) Gaussian filter and bandpass-filtered at the range from 0.01 to 0.08 Hz to remove slow drift and high-frequency components (Biswal et al., 1995; Lowe et al., 1998). No global signal regression was performed to avoid introduction of spurious correlations of the rs-fMRI data (Gotts et al., 2013).

### Amplitude of Low-Frequency Fluctuations and Functional Connectivity Analysis

ALFF analysis was conducted using the rs-fMRI data analysis toolkit v1.1 (http://www.restfmri.net/forum/rest_v11) in MATLAB. For each individual's rs-fMRI data, the filtered time series of each voxel was transformed to the frequency domain to obtain the power spectrum using a fast Fourier transformation (taper percent = 0, FFT length = shortest). The ALFF was derived from the averaged square root of the power spectrum across frequencies from 0.01 to 0.08 Hz (Zang et al., 2007). The ALFF of each voxel was then normalized by the averaged ALFF value of the whole brain.

Seed ROIs for FC analysis were identified using data from the subset of participants (10 individuals with ASD and 11 controls) who completed both rs-fMRI and tb-fMRI runs by determining clusters with significant correlations between ALFF and sustained force variability separately for each group. Monte Carlo simulation was used to correct for multiple comparisons (Ledberg et al., 1998). Based on AlphaSim calculations, clusters including ≥90 contiguous voxels showing significant correlations with *p* < 0.05 at voxel level were identified and are reported to maintain family-wise *p* < 0.05.

FC analysis was performed based on rs-fMRI of all participants using a seed-based voxel correlation approach (Hull et al., 2017). Three dimensional 6-mm-radius seeds were created based on selected ROIs using the PickAtlas toolbox in SPM8 (http://fmri.wfubmc.edu/software/PickAtlas). The center of the sphere for each identified voxel cluster was the coordinate with the greatest correlation between motor variability and ALFF value. To quantify whole-brain connectivity of identified seeds, individual time series for each ROI were extracted and correlated with each voxel in the brain to create a whole brain connectivity map. Each participant's correlation map was then converted to z-statistic maps using Fisher r-to-z transformations.

### Statistical Analyses

One-way ANOVAs were conducted to compare groups on force variability. For imaging data, statistical analyses were conducted using rs-fMRI data analysis toolkit V1.8 (http://www.restfmri.net/forum/REST_V1.8) in MATLAB (Zang et al., 2007). ROIs identified as significantly associated with force variability in the ASD or control group were merged. Then, the maximal ALFF value within each ROI was extracted from each individual and compared between patients and controls using two-sample *t*-tests with the SPSS 22 software (Armonk, NY, USA). For voxel-based comparisons of FC correlation maps between groups, two-sample *t*-tests was used with REST_V1.8 software in Matlab. Age and sex were included as covariates for both statistical analyses. A default mask (dimension = 61 × 73 × 61) was applied for all statistical analyses. Additionally, for individuals with ASD, we examined the relationship between strength of FC for networks that showed significant between-group differences and IQ scores and clinical ratings of social–communication deficits (i.e., ADOS social communication algorithm score). Statistical thresholds for correlation analyses were set at *p* < 0.05 (two-tailed) after false discovery rate (FDR) correction. To inform hypotheses of future studies, uncorrected correlation *r* and *p*-values are also presented in Supplementary Tables 3–7.

## Results

### Visuomotor Behavioral Measure

Individuals with ASD and TD controls showed similar MVCs (*t*_11.43_ = 0.41, *p* = 0.69; ASD: mean = 55.70 N, SD = 20.68 N; TD: mean = 52.82 N, SD = 8.01 N) and mean force (*t*_12.12_ = 0.18, *p* = 0.86; ASD: mean = 31.46 N, SD = 10.58 N; TD: mean = 30.81 N, SD = 4.67 N). The difference in sustained force variability for individuals with ASD and TD controls was not statistically significant (*t*_9.59_ = 1.68, *p* = 0.13; ASD: mean = 3.38 N, SD = 4.07 N; TD: mean = 1.19 N, SD = 0.77 N). While this group comparison was not significant as we have seen in our prior studies (Mosconi et al., 2015; Wang et al., 2015), the effect size was large (Cohen's *d* = 0.75).

### Relationships Between ALFF and Sustained Force Variability

**Figure 2:**
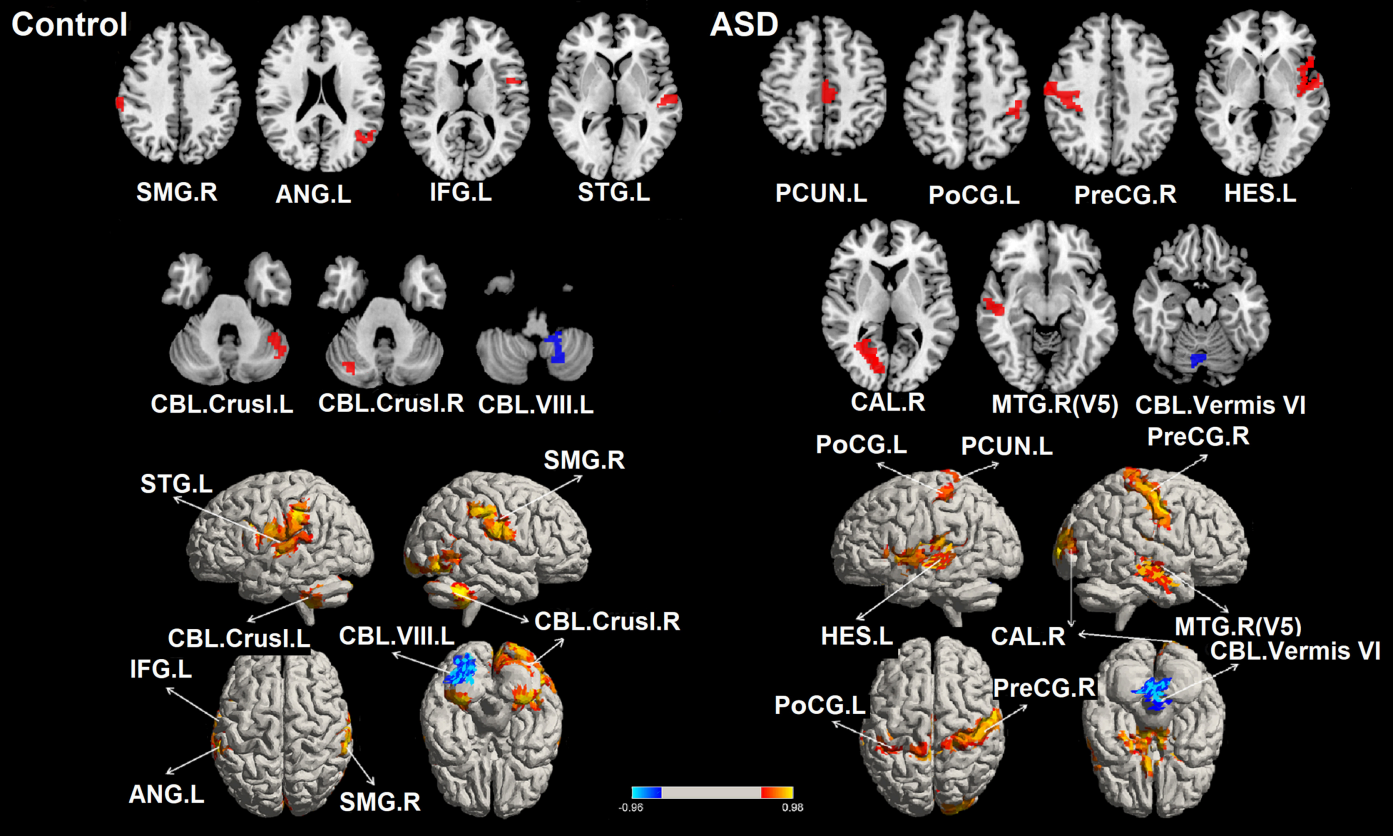
Significant correlations between the whole brain amplitude of low-frequency fluctuations (ALFFs) and sustained force standard deviation in individuals with ASD and TD controls. The red areas depict voxels presenting positive correlations, whereas the blue areas depict voxels showing negative correlations with sustained force standard deviation. IFG.L, left inferior frontal gyrus; PreCG.R, right precentral gyrus; PoCG.L, left postcentral gyrus; SMG.R, right supramarginal gyrus; ANG.L, left angular gyrus; PCUN.L, left precuneus; HES.L, left Heschl's gyrus; STG.L, left superior temporal gyrus; MT/V5. R, right middle temporal gyrus including visual area 5; CAL.R, right calcarine cortex; CBL.Vermis VI, cerebellar vermis VI; CBL.Crus I. L, left cerebellar crus I; CBL.Crus I. R, right cerebellar crus I; CBL. VIII. L, left cerebellar lobule VIII.

Seven ROIs in each group showed significant correlations between whole brain voxel-wise ALFF measures and sustained force variability (Table 2 and Figure 2). ROIs that were positively correlated with force variability for TD controls after corrections for multiple comparisons included left inferior frontal gyrus, right supramarginal gyrus, left angular gyrus, left superior temporal gyrus, and bilateral cerebellar crus I. Left cerebellar lobule VIII ALFF was negatively correlated with force variability for controls.

**Table 2:** Montreal neurological institute (MNI) coordinates of selected seed regions of interest (ROIs) which showed significant correlations between amplitude of low frequency fluctuations (ALFF) and sustained force standard deviation during precision grip in TD controls and individuals with ASD.

**ROIs**	**MNI coordinates**			**Number of voxels**	**Mean (SE)**	**Correlation coefficient *Z***
	**X**	**Y**	**Z**			
**ROIs identified from controls**						
Left inferior frontal gyrus	42	15	24	110	−0.13 (0.10)	0.94^*^
Right supramarginal gyrus	66	−27	39	103	−0.03 (0.06)	0.93^*^
Left angular gyrus	−54	−60	30	180	0.06 (0.07)	0.90^*^
Left superior temporal gyrus	−57	−18	3	31	−0.13 (0.10)	0.95^*^
Left cerebellar crus I	−45	−48	−33	190	0.04(0.13)	0.94^*^
Right cerebellar crus I	24	−78	−27	612	0.17 (0.17)	0.97^*^
Left cerebellar lobule VIII	21	−57	48	147	−0.08 (0.04)	−0.90^*^
**ROIs identified from individuals with ASD**						
Right precentral gyrus	42	−18	42	391	−0.08 (0.05)	0.94^*^
Left postcentral gyrus	−33	−24	45	113	0.01 (0.05)	0.93^*^
Left precuneus	−6	−36	57	241	−0.07 (0.06)	0.94^*^
Left Heschl's gyrus	−45	−15	9	369	−0.00 (0.08)	0.98^*^
Right middle temporal gyrus	60	−15	−9	250	−0.11 (0.11)	0.94^*^
Right calcarine cortex	15	−57	15	342	−0.25 (0.13)	0.94^*^
Cerebellar vermis VI	6	−69	−15	306	−0.05 (0.11)	−0.96^*^

*Negative X values indicate locations in the left hemisphere*.

All results are AlphaSim error corrected,

† p < 0.05,

* p < 0.01,

** p < 0.005,

*** *p < 0.001*.

For individuals with ASD, ALFF levels of the right precentral gyrus, left post-central gyrus, left precuneus, left Heschl's gyrus, right middle temporal gyrus (including MT/V5), and right calcarine cortex were positively correlated with force variability. ALFF levels in the cerebellar vermis VI were negatively correlated with increased force variability in ASD. After FDR correction, none of these 14 ROIs showed between group differences in ALFF (all corrected *p'*s < 0.05; Supplementary Table 2).

### Functional Connectivity in Individuals With ASD and TD Controls

**Figure 3:**
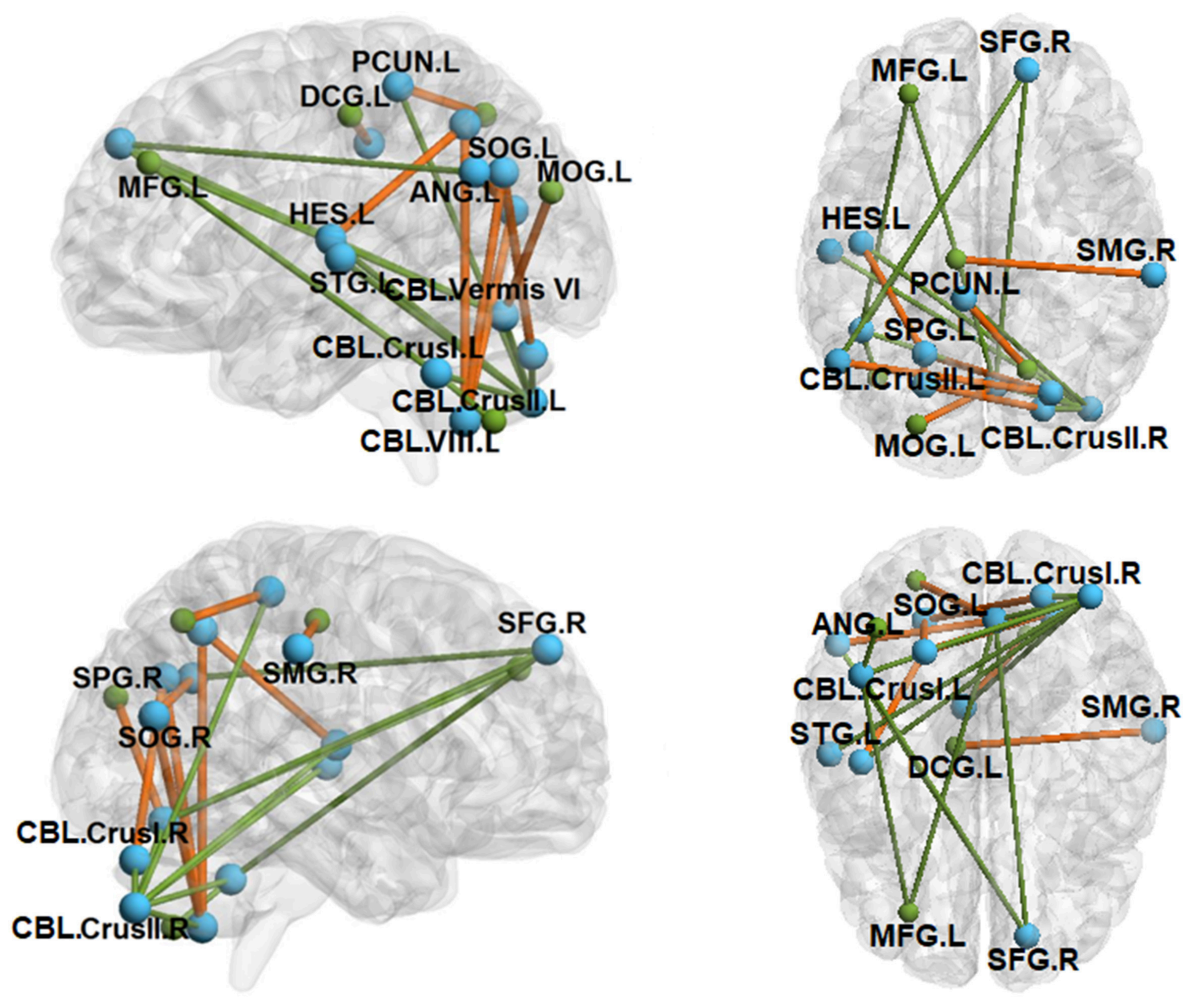
Between-group functional connectivity (FC) maps (ASD vs. TD). Green lines depict FC reductions in individuals with ASD relative to TD controls, while yellow lines represent FC elevations in ASD relative to TD controls. SFG.R, right superior frontal gyrus; MFG.L, left middle frontal gyrus; SPG.L, left superior parietal gyrus; SPG.R, right superior parietal gyrus; SMG.R, right supramarginal gyrus; ANG.L, left angular gyrus; PCUN.L, left precuneus; HES.L, left Heschl's gyrus; STG.L, left superior temporal gyrus; SOG.L, left superior occipital gyrus; SOG.R, right superior occipital gyrus; MOG.L, left middle occipital gyrus; DCG.L, left median cingulate gyrus; CBL.Vermis VI, cerebellar vermis VI; CBL.Crus I.L, left cerebellar crus I; CBL.Crus I.R, right cerebellar crus I; CBL.Crus II.L, left cerebellar crus II; CBL.Crus II.R, right cerebellar crus II; CBL.VIII.L, left cerebellar lobule VIII. All clusters were AlphaSim corrected and statistically significant at *p* < 0.05.

Intra-cerebellar FC was reduced across multiple lobules in ASD (Table 3 and Figure 3). Compared to TD controls, individuals with ASD showed decreased FC between right cerebellar crus II and seeds within left cerebellar lobule VIII, bilateral cerebellar crus I, and cerebellar vermis VI. Individuals with ASD also showed reduced FC between left cerebellar crus I and right cerebellar lobule IX, left cerebellar crus II, and cerebellar vermis VI.

**Table 3:** Between group comparison of functional connectivity (FC) between ROIs identified in ALFF analyses and whole-brain data (positive *T*-values represent greater FC in ASD vs. TD controls).

**ROIs**	**Brain regions**	**MNI coordinates**			**Number of voxels**	***T*-value**
		**X**	**Y**	**Z**		
**ROIs identified from TD controls**						
Right supramarginal gyrus	Left middle cingulate gyrus	−9	−21	48	93	4.00^***^
Left angular gyrus	Right superior frontal gyrus	18	51	39	123	−5.05^***^
	Right superior occipital gyrus	27	−72	18	184	5.04^***^
Left superior temporal gyrus	Right cerebellar crus II	42	−78	−42	168	−4.20^***^
Left cerebellar crus I	Right superior frontal gyrus	27	48	42	92	−4.58^***^
	Left middle frontal gyrus	−27	42	33	110	−3.95^***^
	Left cerebellar crus II	−39	−66	−48	198	−4.92^***^
	Right cerebellar crus II	51	−63	−45	288	−4.96^***^
	Right cerebellar lobule IX	12	−48	−48	120	−3.79^***^
Right cerebellar crus I	Left superior occipital gyrus	−12	−84	18	175	4.65^***^
	Right cerebellar crus II	42	−81	−42	160	−4.08^***^
Left cerebellar lobule VIII	Left superior parietal gyrus	−21	−57	45	159	4.62^***^
	Left superior occipital gyrus	−21	69	30	141	4.47^***^
	Right superior occipital gyrus	27	−69	33	209	4.97^***^
	Right cerebellar crus II	42	−78	−42	137	−3.70^**^
**ROIs identified from individuals with ASD**						
Left precuneus	Right superior parietal gyrus	18	−63	48	143	4.36^***^
	Right cerebellar crus II	6	−84	−39	92	−3.67^**^
Left Heschl's gyrus	Left superior parietal gyrus	−24	−39	48	101	3.96^***^
	Right cerebellar crus II	21	−81	−57	105	−3.75^**^
Cerebellar vermis VI	Right superior frontal gyrus	24	57	15	187	−5.20^***^
	Left middle frontal gyrus	−27	45	36	138	−3.94^***^
	Left middle occipital gyrus	−24	−84	24	102	4.49^***^
	Left cerebellar crus I	−45	−57	−39	277	−4.93^***^
	Right cerebellar crus II	42	−81	−42	241	−4.42^***^

*Negative X-values indicate locations in the left hemisphere*.

All results are AlphaSim corrected,

† p < 0.05,

* p < 0.01,

** p < 0.005,

*** *p < 0.001*.

Cerebellar FC with pre-frontal and temporal cortical targets was reduced in ASD relative to TD controls. Individuals with ASD showed decreased FC between right cerebellar crus II and both left Heschl's gyrus and left superior temporal gyrus. For left cerebellar crus I and cerebellar vermis VI, individuals with ASD showed lower FC than controls with left middle frontal gyrus and right superior frontal gyrus.

Cerebellar FC with occipital and parietal cortical targets was primarily elevated in ASD relative to controls. Individuals with ASD showed increased FC between left cerebellar lobule VIII and bilateral superior occipital gyrus and left superior parietal gyrus. FC between right cerebellar crus I and left superior occipital gyrus was also increased in ASD relative to controls. Right cerebellar crus II FC with left precuneus was reduced in ASD compared to controls.

### Demographic and Clinical Correlations

After FDR correction, no significant correlations were found between ALFF values of our 14 selected ROIs and IQ scores for healthy controls (Supplementary Table 3) or individuals with ASD (Supplementary Table 4). No ROI ALFF values were related to ASD clinical ratings for patients (Supplementary Table 4). No significant correlations were identified between each cortical–cerebellar FC and IQ scores (Supplementary Table 5) or ADOS social communication algorithm scores for individuals with ASD (Supplementary Table 6). Increased FC between left precuneus and right superior parietal gyrus was associated with less severe RBS-R rated repetitive behaviors in ASD. No significant correlations were identified between FC of cortical–cerebellar networks and sustained force variability for TD controls (Supplementary Table 7). Greater FC between left cerebellar crus I and right cerebellar lobule IX was associated with reduced sustained force variability in individuals with ASD (Supplementary Table 7).

## Discussion

In the present study, we identify multiple discrete cortical and cerebellar brain regions showing intrinsic functional oscillations that covary with precision visuomotor ability. The pattern of ROIs showing intrinsic functional properties associated with visuomotor variability were distinct for individuals with ASD and TD controls. Specifically, greater ALFF values of sensorimotor cortical and cerebellar brain regions were associated with greater force variability (i.e., less force precision) in ASD, whereas greater ALFF values in cortical and cerebellar brain regions that comprise higher-order association networks were related to greater force variability in TD controls. Further, we find that FC in patients was greater in cerebellar–occipital and cerebellar–parietal circuits associated with fundamental sensory and sensorimotor processes, whereas it was reduced in cerebellar–frontal and cerebellar–temporal cortical circuits involved in more complex multisensory and cognitive processes. Overall, these findings suggest atypical specialization of brain networks associated with visuomotor behavior in ASD.

### Intrinsic Cortical–Cerebellar Activity and Visuomotor Precision

ALFF power during rest reflects intrinsic oscillations of local circuits including changes in synaptic activity (Fox et al., 1988), neurotransmitter recycling (Magistretti and Pellerin, 1999), and synchronous neuronal firing (Logothetis, 2002; Scholvinck et al., 2010). Long-duration rs-fMRI studies of non-human primates have identified strong correlations between local field potentials and fluctuations in BOLD time series from nearby ROIs at both gamma and lower-frequency bands with consistent time lags of 6–8 s (Shmuel and Leopold, 2008), implying a causal relationship between spontaneous neuronal activity and local hemodynamics. Neuroimaging studies in humans have further demonstrated that intrinsic brain activity accounts for a significant proportion of variance (up to 74%; Fox et al., 2007) of tb-BOLD responses and motor behavioral outcomes (Fox et al., 2006, 2007). Intrinsic BOLD oscillations thus represent important signals interacting with task-related neuronal functions to support adaptation to task demands (Lv et al., 2018). Based on these observations, it has been postulated that the relative balance of intrinsic oscillations and task-specific regional activation is critical for guiding precise sensorimotor behaviors.

Our finding that force variability was significantly correlated with distinct cortical and cerebellar brain regions supports this hypothesis. Positive associations between intrinsic cortical activity and force variability suggest that resting activity of sensorimotor networks in ASD serves as “interference” during visuomotor behavior by either reducing signal-to-noise ratios of task-dependent cortical functions or attenuating the extent to which cortical circuits can dynamically adjust to support the onset and maintenance of behavior. Findings that ALFF levels of cerebellar regions (Table 2; i.e., left cerebellar lobule VIII in controls and cerebellar vermis VI in ASD) dedicated to sensorimotor processes were anti-correlated with force variability suggest that reduced intrinsic cerebellar activations are associated with attenuation of inhibitory output that serves to adjust cortical output during continuous sensorimotor behaviors. Cerebellar cortical output *via* Purkinje cells provides an inhibitory drive on cortical targets that supports refinement of motor behavior in response to sensory feedback (Stein and Glickstein, 1992). Cerebellar lobule VIII and vermis VI each innervate sensory and motor cortices, including frontal eye fields, parietal eye fields, inferior and superior parietal lobules, primary sensory cortex (S1), and primary motor cortex (M1) to support control of precision visuomotor activities (Glickstein, 2000; Ramnani, 2006). Our findings suggesting that reduced inhibitory drive on sensory and motor cortices at rest relates to greater motor variability may reflect amplified task-related modulation of sensory and motor cortices during action contributing to greater output variability over time.

Our brain–behavior results implicate independent cortical and cerebellar regions in the control of visuomotor behavior among individuals with ASD and TD controls. Analyses of individuals with ASD showed positive associations between force variability and ALFF of right M1, left S1, left precuneus, right middle temporal gyrus including MT/V5, and striate cortex (V1). V1 and precuneus are primarily involved in processing initial visual inputs, whereas MT/V5 is dedicated to supporting processing of visual motion. Left S1 and right M1 both are actively involved in prehensile movements including finger tapping (Muller et al., 2001; Mostofsky et al., 2009), grasping (Cavina-Pratesi et al., 2010), and precision gripping (Ehrsson et al., 2000; Coombes et al., 2010). While S1 involvement in visuomotor tasks is typically right dominant, M1 activity is typically lateralized to the contralateral (left) hemisphere. Greater left S1 and ipsilateral (right) M1 activation are seen when visuomotor tasks are more difficult (Post et al., 2009), as in our test of precision force at 60% of individuals' maximum output. Additionally, individuals with ASD showed an inverse relationship between motor variability and intrinsic activity of cerebellar vermis VI, a region implicated in the guidance of precision visuomotor behaviors including saccadic and smooth pursuit eye movements (Takarae et al., 2007).

In contrast to individuals with ASD, brain–behavior associations for TD controls implicate regions outside of primary sensorimotor networks and include cortical and cerebellar association circuits. Specifically, left inferior frontal gyrus is involved in visuomotor gripping (Ehrsson et al., 2000) and learning of complex finger tapping sequences (Muller et al., 2002) due to its reciprocal projections with principal sensorimotor areas of premotor cortex, frontal eye fields, and striatum (Husain, 1991). Right supramarginal gyrus supports motor programming through its connections to the paracentral lobule, supplementary motor area, premotor cortex, and insula (Hesse et al., 2006). Angular gyrus is dedicated to attentional processes supporting individuals' engagement during goal-directed behaviors (Arsalidou and Taylor, 2011). Cerebellar crus I activity during basic visuomotor tasks scales with the frequency of visual feedback, suggesting that it is critical to adapting ongoing motor behavior to complex sensory information (Vaillancourt et al., 2006). Important regional variations thus suggest that TD controls' visuomotor behavior may be disrupted by intrinsic cortical–cerebellar activities in association networks involved in more complex processes. In contrast, individuals with ASD appear more susceptible to intrinsic variations affecting primary sensorimotor networks, suggesting a greater reliance on more fundamental neural networks to support the refinement of basic visuomotor behavior.

ALFF frequencies reported here are within the range of delta oscillations (0–4 Hz) associated with force variability during slow isometric force production and neocortical “common drive” modulation of the skeletal motor neuron pool (De Luca et al., 1982a; Lodha and Christou, 2017). Our finding that greater ALFF power in sensorimotor networks is associated with elevated force variability in ASD but not in TD individuals suggests that patients show atypical common drive modulation of neuromuscular systems during rest. In the context of our previous EMG findings documenting reduced delta modulation and reduced linkage between modulation of the motor neuron pool at multiple frequency bands (i.e., delta, beta, and gamma) and force variability in ASD (Wang et al., 2017), our rs-fMRI findings implicate alterations in corticomuscular coherence that contribute to a reduced ability to precisely control force output in patients. Other studies also identified the effect of motor learning on beta and gamma band modulation within the context of individualized differences (Witte et al., 2007; Mendez-Balbuena et al., 2012). As precision visuomotor control is repetitively implicated in individuals with ASD at multiple target force levels (Mosconi et al., 2015; Wang et al., 2015), studies examining corticomuscular coherence at multiple frequency bands using EEG and EMG (Mendez-Balbuena et al., 2012) are warranted.

### Intra-Cerebellar and Cortical–Cerebellar Functional Connectivity in ASD

Relative to TD controls, individuals with ASD showed reduced intrinsic FC between medial cerebellar lobules dedicated to sensorimotor processes (vermis VI, posterior VIII and IX) and lateral lobules involved in higher-order processes (crus I/II), suggesting reduced interactions of distinct functional circuits within the cerebellum (Table 3 and Figure 3). These distinct cerebellar circuits are anatomically and functionally linked to separate cortical targets. Medial lobules innervate dorsomedial thalamic nuclei and motor and parietal cortices, whereas crus I/II are more ontogenetically and phylogenetically advanced and are most densely connected with pre-frontal and association cortices (Ramnani, 2006). During goal-directed activities, cerebellar crus I/II circuits are involved in integrating complex and multi-sensory information (Nitschke et al., 2005; Stoodley and Schmahmann, 2009; D'Mello and Stoodley, 2015). Reduced FC between crus I/II and more medial cerebellar circuits in ASD suggest deficits integrating multisensory information and utilizing higher-level inputs to guide sensorimotor behaviors. Our FC results are consistent with prior DTI studies that show decreased white matter microstructural integrity within the cerebellum and within fiber tracts connecting cerebellar cortex and dentate nucleus in ASD (Sivaswamy et al., 2010; Jeong et al., 2014; Crippa et al., 2016). The cerebellum has also been consistently implicated in histological and MRI studies of ASD that identified reductions in the number and size of Purkinje cells (Bauman, 1991; Courchesne, 1997; Whitney et al., 2008) and hypoplasia of lobules V–VII (Courchesne et al., 1988). Our findings indicate that cerebellar pathology and white matter microstructural variation may be associated with reduced communication between functionally distinct circuits in ASD.

We also documented that cerebellar FC with pre-frontal cortical targets, including right superior and left middle frontal gyri are reduced in ASD, while cerebellar FC with posterior parietal and occipital cortices is elevated (Figure 3). These findings are consistent with prior studies documenting reduced rs-FC between right cerebellar crus I/II and contralateral pre-frontal cortex and inferior/middle temporal gyrus (Khan et al., 2015), and between right crus I and contralateral superior frontal gyrus, middle frontal gyrus, thalamus, anterior cingulate gyrus, and parietal cortex in adolescents with ASD (Verly et al., 2014). Reduced tb-FC of the cerebellum and M1, supplementary motor area, and thalamus was also reported during sequential finger tapping in ASD (Mostofsky et al., 2009). Consistent with findings of reduced FC between cerebellum and cortex, DTI studies of individuals with ASD have documented atypical white matter microstructural integrity of the primary cortical input and output pathways of the cerebellum—the middle and superior peduncles (Catani et al., 2008; Brito et al., 2009; Hanaie et al., 2013). As cerebellar circuits integrate and relay error information to frontal, parietal, and temporal cortices (Glickstein, 2000; D'Mello and Stoodley, 2015), reduced cerebellar FC with the left middle frontal gyrus, right superior frontal gyrus, and contralateral superior temporal gyrus suggests that cerebellar disconnectivity may play a key role in a broad range of neurodevelopmental dysfunctions in ASD, including executive, language, and multisensory processes. Further, greater FC between the cerebellum and occipital and posterior parietal circuits suggests increased reliance on more basic sensory information for guiding behavior, as suggested previously in studies of motor learning (Haswell et al., 2009; Izawa et al., 2012).

### Limitations and Future Directions

One limitation of the present study is the small sample of individuals who completed both tb- and rs-fMRI runs. However, given the large magnitudes of correlations between selected ROIs and sustained force variability (Table 2), our results appear to be robust. Additional analyses across larger samples will be important for determining how these brain–behavior relationships vary across the broader ASD population. Future studies may also examine separate measures of intrinsic brain activity in relation to behavioral issues in ASD. For example, recent studies have quantified the dynamics of intrinsic brain activity using the variance of ALFF over time (Li et al., 2018; Liao et al., 2019). While we focused on mean ALFF across the time series based on the strong relationships previously demonstrated between mean ALFF and sensorimotor behavior (Ma et al., 2011), examination of temporal variability may provide key insights into neural mechanisms. Future studies should also examine the relationships between intrinsic activity of sensorimotor networks and behavior across both hands, as lateralized deficits of sensorimotor behavior and brain function have been identified in ASD (Kleinhans et al., 2008). As we have previously found that the severity of visuomotor deficits in ASD varies as a function of visual feedback gain and force level (Mosconi et al., 2015), determining the extent to which brain–behavior linkages vary across different levels of visual feedback and force load will also be important for future studies. Finally, analysis of FC of cerebellar–cortical systems in relation to DTI data quantifying white matter microstructural integrity alterations in these networks in ASD may provide important new insights into mechanisms associated with sensorimotor network dysfunctions and elevations in motor variability.

## Conclusions

The current work demonstrates that intrinsic neural oscillations in sensorimotor cortical and cerebellar circuits are strongly associated with visuomotor precision in both individuals with ASD and TD controls. We also show important regional and network dissociations of the intrinsic functional anatomy of visuomotor control in ASD and TD. Our findings of reduced intracerebellar, cerebellar–frontal, and cerebellar–temporal FC in ASD suggest that previously documented pathologies of the cerebellum may interfere with multiple developmental functions involving both basic sensorimotor and higher-order association networks. As disruptions of basic sensorimotor processes involving cortical–cerebellar circuits are seen in ASD across the lifespan, focus on intrinsic functional properties of these networks may provide important insights into neurodevelopmental processes that interfere with both early emerging and more complex behaviors.

## Ethics Statement

All study procedures were approved by the Institutional Review Boards at the UT Southwestern Medical Center and Children's Hospital of Dallas. Adult participants provided both informed and written consent, and minors provided assent in addition to informed and written consent from their legal guardian.

## Author Contributions

MM and JS are responsible for the conception and design of the research. ZW scored and analyzed the behavioral data. MM, JS, QG, and SL supervised YW to score and analyze the neuroimaging data. ZW and YW performed statistical analyses. ZW, YW, SL, QG, JS, and MM interpreted the experimental results. ZW and YW prepared figures and tables and drafted the manuscript. Each author edited the manuscript. All authors have approved the final version of the manuscript.

### Conflict of Interest Statement

ZW serves as a co-investigator on an investigator-initiated award studying Phelan-McDermid Syndrome from Novartis. JS serves on the advisory boards for Roche, Takeda, and Eli Lilly. The authors declare that the research was conducted in the absence of any commercial or financial relationships that could be construed as a potential conflict of interest.

## Abbreviations

ASD: autism spectrum disorder
tb-fMRI: task-based functional MRI
rs-fMRI: resting-state functional MRI
BOLD: blood-oxygen-level dependent
FC: functional connectivity
ROI: region of interest
ALFF: amplitude of low-frequency fluctuations
FSIQ: full scale IQ
PIQ: performance IQ
VIQ: verbal IQ
RBS-R: repetitive behaviors scale-revised
SFG.R: right superior frontal gyrus
MFG.L: left middle frontal gyrus
IFG.L: left inferior frontal gyrus
Pre-CG.R: right pre-central gyrus
PoCG.L: left post-central gyrus
SPG.L: left superior parietal gyrus
SPG.R: right superior parietal gyrus
SMG.R: right supramarginal gyrus
ANG.L: left angular gyrus
PCUN.L: left precuneus
HES.L: left Heschl's gyrus
STG.L: left superior temporal gyrus
MT/V5. R: right middle temporal gyrus including visual area 5
SOG.R: right superior occipital gyrus
SOG.L: left superior occipital gyrus
MOG.L: left middle occipital gyrus
CAL.R: right calcarine cortex
DCG.L: left median cingulate gyrus
CBL.Vermis VI: cerebellar vermis VI
CBL.Crus I.L: left cerebellar crus I
CBL.Crus I.R: right cerebellar crus I
CBL.Crus II.L: left cerebellar crus II
CBL.Crus II.R: right cerebellar crus II
CBL.VIII.L: left cerebellar lobule VIII.
