# Supplementary material for: Resting-State Brain Network Dysfunctions Associated With Visuomotor Impairments in Autism Spectrum Disorder
Source: Front Integr Neurosci. 2019 May 31;13:17. doi: 10.3389/fnint.2019.00017 (PMC6554427; doi:10.3389/fnint.2019.00017)
Supplement: Supplementary file 1 [file Table_1.docx]

**Supplementary information**

**Supplementary Table 1 (sT1)**

**Between group comparisons (ASD vs. TD Controls) of translational (Tx,Ty,Tz) and rotational (Rx,Ry,Rz) head motion parameters. No head motion parameters showed significant differences between groups**

| **Motion parameters** | **t** | **P** | **Mean (SE)** |
| --- | --- | --- | --- |
| Tx(mm) | 0.75 | 0.46 | 0.02 (0.03) |
| Ty(mm) | -0.01 | 0.99 | 0.00 (0.04) |
| Tz(mm) | 0.48 | 0.63 | 0.02 (0.04) |
| Rx(º) | 0.62 | 0.53 | 0.04 (0.07) |
| Ry(º) | 0.52 | 0.61 | 0.02 (0.04) |
| Rz(º) | 0.61 | 0.54 | 0.02 (0.04) |

All results are FDR corrected, †p < 0.05, ∗p < 0.01, ∗∗p < 0.005, ∗∗∗p < 0.001

Positive t value indicates ASD> TD Controls, negative t value indicates ASD< TD Controls
