# Supplementary material for: Resting-State Brain Network Dysfunctions Associated With Visuomotor Impairments in Autism Spectrum Disorder
Source: Front Integr Neurosci. 2019 May 31;13:17. doi: 10.3389/fnint.2019.00017 (PMC6554427; doi:10.3389/fnint.2019.00017)
Supplement: Supplementary file 2 [file Table_2.docx]

**Supplementary Table 2 (sT2)**

**Between group comparisons (ASD vs. TD Controls) of ALFF of 14 selected regions of interest (ROIs). No ROIs showed significant differences between groups**

| **ROIs** | **t** | **P** | **Mean (SE)** |
| --- | --- | --- | --- |
| **Left inferior frontal gyrus** | **0.81** | **0.42** | **0.06 (0.08)** |
| Right precentral gyrus | -1.62 | 0.11 | -0.08 (0.05) |
| Left postcentral gyrus | 0.14 | 0.89 | 0.01 (0.05) |
| Right supramarginal gyrus | -0.58 | 0.57 | -0.03 (0.06) |
| Left angular gyrus | 0.95 | 0.35 | 0.06 (0.07) |
| Left precuneus | -1.22 | 0.23 | -0.07 (0.06) |
| Left Heschl’s gyrus | -0.02 | 0.99 | -0.00 (0.08) |
| Left superior temporal gyrus | -1.35 | 0.19 | -0.13 (0.10) |
| Right middle temporal gyrus | -1.01 | 0.32 | -0.11 (0.11) |
| Right calcarine cortex | -1.96 | 0.06 | -0.25 (0.13) |
| Cerebellar vermis VI | -0.46 | 0.65 | -0.05 (0.11) |
| Left cerebellar crus I | 0.31 | 0.76 | 0.04 (0.13) |
| Right cerebellar crus I | 1.03 | 0.31 | 0.17 (0.17) |
| Left cerebellar lobule VIII | -1.97 | 0.06 | -0.08 (0.04) |

All results are FDR corrected, †p < 0.05, ∗p < 0.01, ∗∗p < 0.005, ∗∗∗p < 0.001

Positive t value indicates ASD> TD Controls, negative t value indicates ASD< TD Controls
