# Supplementary material for: Resting-State Brain Network Dysfunctions Associated With Visuomotor Impairments in Autism Spectrum Disorder
Source: Front Integr Neurosci. 2019 May 31;13:17. doi: 10.3389/fnint.2019.00017 (PMC6554427; doi:10.3389/fnint.2019.00017)
Supplement: Supplementary file 3 [file Table_3.docx]

**Supplementary Table 3 (sT3)**

**Correlations between amplitude of low frequency fluctuation (ALFF) of selected ROIs and IQ scores for TD controls**

|  | **Full scale IQ** | | | **Performance IQ** | | | **Verbal IQ** | | |
| --- | --- | --- | --- | --- | --- | --- | --- | --- | --- |
|  | **r** | **P** | **FDR** | **r** | **P** | **FDR** | **r** | **P** | **FDR** |
| Left inferior frontal gyrus | 0.00 | 0.99 | 0.99 | -0.21 | 0.44 | 0.92 | 0.15 | 0.60 | 0.92 |
| Right precentral gyrus | 0.08 | 0.79 | 0.92 | 0.14 | 0.62 | 0.92 | 0.03 | 0.93 | 0.97 |
| Left postcentral gyrus | 0.26 | 0.34 | 0.92 | 0.15 | 0.60 | 0.92 | 0.30 | 0.28 | 0.92 |
| Right supramarginal gyrus | -0.02 | 0.94 | 0.97 | -0.10 | 0.73 | 0.92 | 0.06 | 0.84 | 0.94 |
| Left angular gyrus | 0.09 | 0.76 | 0.92 | -0.08 | 0.77 | 0.92 | 0.21 | 0.45 | 0.92 |
| Left precuneus | -0.27 | 0.34 | 0.92 | -0.22 | 0.43 | 0.92 | -0.26 | 0.34 | 0.92 |
| Left Heschl’s gyrus | -0.05 | 0.85 | 0.94 | 0.11 | 0.71 | 0.92 | -0.17 | 0.55 | 0.92 |
| Left superior temporal gyrus | -0.03 | 0.91 | 0.97 | -0.28 | 0.32 | 0.92 | 0.15 | 0.61 | 0.92 |
| Right middle temporal gyrus | -0.43 | 0.11 | 0.92 | -0.60 | 0.02^†^ | 0.76  0 | -0.23 | 0.42 | 0.92 |
| Right calcarine cortex | -0.20 | 0.47 | 0.92 | -0.19 | 0.49 | 0.92 | -0.13 | 0.65 | 0.92 |
| Cerebellar vermis VI | -0.42 | 0.12 | 0.92 | -0.54 | 0.04^†^ | 0.82 | -0.26 | 0.34 | 0.92 |
| Left cerebellar crus I | 0.08 | 0.78 | 0.92 | -0.13 | 0.64 | 0.92 | 0.19 | 0.50 | 0.92 |
| Right cerebellar crus I | -0.16 | 0.57 | 0.92 | -0.46 | 0.09 | 0.92 | 0.08 | 0.79 | 0.92 |
| Left cerebellar lobule VIII | 0.23 | 0.41 | 0.92 | 0.35 | 0.21 | 0.92 | 0.12 | 0.67 | 0.92 |

Statistical significance *before* FDR correction, †p < 0.05
