# Supplementary material for: Resting-State Brain Network Dysfunctions Associated With Visuomotor Impairments in Autism Spectrum Disorder
Source: Front Integr Neurosci. 2019 May 31;13:17. doi: 10.3389/fnint.2019.00017 (PMC6554427; doi:10.3389/fnint.2019.00017)
Supplement: Supplementary file 4 [file Table_4.docx]

**Supplementary Table 4 (sT4)**

**Correlations between ALFF of selected ROIs, IQ scores and clinical ratings for individuals with ASD**

|  | **Full scale IQ** | | | **Performance IQ** | | | **Verbal IQ** | | | | **ADOS Social** | | | | **RBS-R Total** | | | |  |
| --- | --- | --- | --- | --- | --- | --- | --- | --- | --- | --- | --- | --- | --- | --- | --- | --- | --- | --- | --- |
|  | **r** | **P** | **FDR**  **R** | **r** | **P** | **FDR** | | **r** | **P** | **FDR** | | **r** | **P** | **FDR** | | **r** | **P** | **FDRR** | |
| Left inferior frontal gyrus | -0.13 | 0.55 | 0.94 | -0.13 | 0.55 | 0.94 | | -0.11 | 0.61 | 0.94 | | 0.10 | 0.66 | 0.94 | | 0.00 | 0.99 | 0.99 | |
| Right precentral gyrus | -0.11 | 0.62 | 0.94 | -0.13 | 0.57 | 0.94 | | -0.07 | 0.76 | 0.94 | | -0.33 | 0.14 | 0.89 | | -0.28 | 0.23 | 0.89 | |
| Left postcentral gyrus | -0.01 | 0.95 | 0.99 | 0.08 | 0.73 | 0.94 | | -0.09 | 0.70 | 0.94 | | 0.04 | 0.85 | 0.94 | | -0.06 | 0.81 | 0.94 | |
| Right supramarginal gyrus | 0.04 | 0.86 | 0.94 | 0.15 | 0.51 | 0.93 | | -0.08 | 0.73 | 0.94 | | -0.04 | 0.85 | 0.94 | | 0.10 | 0.69 | 0.94 | |
| Left angular gyrus | 0.05 | 0.82 | 0.94 | 0.01 | 0.97 | 0.99 | | 0.05 | 0.82 | 0.94 | | -0.09 | 0.69 | 0.94 | | -0.65 | .002^**^* | 0.14 | |
| Left precuneus | -0.24 | 0.28 | 0.89 | -0.08 | 0.73 | 0.94 | | -0.33 | 0.13 | 0.89 | | 0.24 | 0.29 | 0.89 | | -0.08 | 0.75 | 0.94 | |
| Left Heschl’s gyrus | -0.24 | 0.27 | 0.89 | -0.17 | 0.43 | 0.92 | | -0.24 | 0.27 | 0.89 | | 0.04 | 0.85 | 0.94 | | -0.51 | 0.02^†^ | 0.74 | |
| Left superior temporal gyrus | 0.28 | 0.19 | 0.89 | 0.16 | 0.46 | 0.92 | | 0.32 | 0.14 | 0.89 | | -0.19 | 0.39 | 0.91 | | -0.35 | 0.13 | 0.89 | |
| Right middle temporal gyrus | -0.24 | 0.28 | 0.89 | -0.21 | 0.33 | 0.89 | | -0.22 | 0.31 | 0.89 | | -0.39 | 0.08 | 0.89 | | -0.22 | 0.34 | 0.89 | |
| Right calcarine cortex | -0.18 | 0.40 | 0.91 | -0.18 | 0.40 | 0.91 | | -0.15 | 0.49 | 0.92 | | -0.04 | 0.85 | 0.94 | | -0.37 | 0.11 | 0.89 | |
| Cerebellar vermis VI | 0.28 | 0.20 | 0.89 | 0.17 | 0.44 | 0.92 | | 0.32 | 0.14 | 0.89 | | 0.00 | 0.99 | 0.99 | | 0.08 | 0.75 | 0.94 | |
| Left cerebellar crus I | -0.12 | 0.59 | 0.94 | -0.03 | 0.90 | 0.97 | | -0.15 | 0.49 | 0.92 | | 0.25 | 0.26 | 0.89 | | 0.17 | 0.47 | 0.92 | |
| Right cerebellar crus I | 0.25 | 0.26 | 0.89 | 0.28 | 0.20 | 0.89 | | 0.20 | 0.37 | 0.91 | | 0.02 | 0.94 | 0.99 | | 0.24 | 0.32 | 0.89 | |
| Left cerebellar lobule VIII | 0.34 | 0.12 | 0.89 | 0.35 | 0.10 | 0.89 | | 0.22 | 0.31 | 0.89 | | -0.12 | 0.59 | 0.94 | | -0.14 | 0.54 | 0.94 | |

ADOS Social= ADOS Social: ADOS social communication domain affect score; RBS-R total: repetitive behaviors scale-revised total score

Statistical significance *before* FDR correction, †p < 0.05, ∗p < 0.01, ∗∗p < 0.005, ∗∗∗p < 0.001
