# Supplementary material for: Resting-State Brain Network Dysfunctions Associated With Visuomotor Impairments in Autism Spectrum Disorder
Source: Front Integr Neurosci. 2019 May 31;13:17. doi: 10.3389/fnint.2019.00017 (PMC6554427; doi:10.3389/fnint.2019.00017)
Supplement: Supplementary file 5 [file Table_5.docx]

**Supplementary Table 5 (sT5)**

**Correlations between functional connectivity (FC)** **which significantly differed between groups and IQ scores for individuals with ASD**

|  | **Full scale IQ** | | | **Performance IQ** | | | **Verbal IQ** | | |
| --- | --- | --- | --- | --- | --- | --- | --- | --- | --- |
|  | **r** | **P** | **FDR** | **r** | **P** | **FDR** | **r** | **P** | **FDR** |
| SMG.R-DCG.L | -0.01 | 0.98 | 0.99 | -0.08 | 0.72 | 0.99 | 0.05 | 0.83 | 0.99 |
| ANG.L-SFG.R | 0.23 | 0.30 | 0.99 | 0.40 | 0.06 | 0.66 | 0.02 | 0.94 | 0.99 |
| ANG.L-SOG.R | -0.20 | 0.35 | 0.99 | -0.17 | 0.45 | 0.99 | -0.16 | 0.48 | 0.99 |
| PCUN.L-SPG.R | 0.08 | 0.73 | 0.99 | 0.14 | 0.54 | 0.99 | 0.02 | 0.94 | 0.99 |
| PCUN.L-CBL.Crus II. R | 0.52 | 0.01^†^ | 0.40 | 0.55 | 0.01^*^ | 0.40 | 0.42 | 0.05† | 0.66 |
| HES.L-SPG.L | 0.02 | 0.93 | 0.99 | 0.05 | 0.82 | 0.99 | 0.02 | 0.94 | 0.99 |
| HES.L-CBL.Crus II. R | -0.17 | 0.43 | 0.99 | -0.15 | 0.48 | 0.99 | -0.16 | 0.48 | 0.99 |
| STG.L-CBL.Crus II. R | -0.37 | 0.08 | 0.71 | -0.26 | 0.23 | 0.99 | -0.39 | 0.06 | 0.66 |
| CBL.Vermis VI-SFG.R | -0.07 | 0.75 | 0.99 | -0.02 | 0.93 | 0.99 | -0.11 | 0.63 | 0.99 |
| CBL.Vermis VI-MFG.L | -0.10 | 0.64 | 0.99 | -0.15 | 0.51 | 0.99 | -0.04 | 0.86 | 0.99 |
| CBL.Vermis VI-MOG.L | -0.01 | 0.95 | 0.99 | -0.13 | 0.56 | 0.99 | 0.11 | 0.61 | 0.99 |
| CBL.Vermis VI-CBL.Crus I. L | 0.00 | 0.99 | 0.99 | -0.04 | 0.87 | 0.99 | 0.01 | 0.97 | 0.99 |
| CBL.Vermis VI-CBL.Crus II. R | -0.24 | 0.26 | 0.99 | -0.15 | 0.50 | 0.99 | -0.25 | 0.25 | 0.99 |
| CBL.Crus I.L-SFG.R | 0.40 | 0.06 | 0.66 | 0.30 | 0.16 | 0.99 | 0.40 | 0.06 | 0.66 |
| CBL.Crus I.L-MFG.L | 0.10 | 0.65 | 0.99 | 0.12 | 0.60 | 0.99 | 0.05 | 0.83 | 0.99 |
| CBL.Crus I. L-CBL.Crus II. L | 0.21 | 0.34 | 0.99 | 0.10 | 0.65 | 0.99 | 0.23 | 0.29 | 0.99 |
| CBL.Crus I. L-CBL.Crus II. R | -0.26 | 0.23 | 0.99 | -0.27 | 0.22 | 0.99 | -0.20 | 0.35 | 0.99 |
| CBL.Crus I. L-CBL. IX. R | -0.27 | 0.21 | 0.99 | -0.18 | 0.41 | 0.99 | -.029 | 0.18 | 0.99 |
| CBL.Crus I.R-SOG.L | 0.01 | 0.98 | 0.99 | 0.17 | 0.44 | 0.99 | -0.11 | 0.62 | 0.99 |
| CBL.Crus I. R-CBL.Crus II. R | -0.22 | 0.31 | 0.99 | -0.08 | 0.71 | 0.99 | -0.27 | 0.22 | 0.99 |
| CBL. VIII. L-SPG.L | -0.16 | 0.47 | 0.99 | 0.01 | 0.96 | 0.99 | -.027 | 0.21 | 0.99 |
| CBL. VIII. L-SOG.L | 0.02 | 0.94 | 0.99 | -0.01 | 0.97 | 0.99 | 0.03 | 0.90 | 0.99 |
| CBL. VIII. L-SOG.R | 0.02 | 0.93 | 0.99 | -0.04 | 0.85 | 0.99 | 0.05 | 0.82 | 0.99 |
| CBL. VIII. L-CBL.Crus II. R | -0.12 | 0.57 | 0.99 | -0.01 | 0.97 | 0.99 | -0.20 | 0.35 | 0.99 |

SFG.R= right superior frontal gyrus; MFG.L= left middle frontal gyrus; SPG.L= left superior parietal gyrus;SPG.R= right superior parietal gyrus; SMG.R=right supramarginal gyrus; ANG.L= left angular gyrus; PCUN.L=left precuneus; HES.L=left Heschl's gyrus; STG.L=left superior temporal gyrus; SOG.L= left superior occipital gyrus; SOG.R=right superior occipital gyrus; MOG.L= left middle occipital gyrus; DCG.L= left median cingulate gyrus; CBL.Vermis VI=cerebellar vermis VI; CBL.Crus I.L=left cerebellar crus I; CBL.Crus I.R=right cerebellar crus I; CBL.Crus II.L=left cerebellar crus II; CBL.Crus II.R=right cerebellar crus II; CBL.VIII.L=left cerebellar lobule VIII.

Statistical significance *before* FDR correction, †p < 0.05, ∗p < 0.01, ∗∗p < 0.005, ∗∗∗p < 0.001
