# Supplementary material for: Resting-State Brain Network Dysfunctions Associated With Visuomotor Impairments in Autism Spectrum Disorder
Source: Front Integr Neurosci. 2019 May 31;13:17. doi: 10.3389/fnint.2019.00017 (PMC6554427; doi:10.3389/fnint.2019.00017)
Supplement: Supplementary file 6 [file Table_6.docx]

**Supplementary Table 6 (sT6)**

**Correlations between functional connectivity (FC) which significantly differed between groups and clinical ratings for individuals with ASD**

|  | **ADOS Social** | | | **RBS-R Total** | | |  |
| --- | --- | --- | --- | --- | --- | --- | --- |
|  | **r** | **P** | **FDR** | **r** | **P** | **FDR** |  |
| SMG.R-DCG.L | 0.13 | 0.57 | 0.97 | -0.14 | 0.55 | 0.97 |  |
| ANG.L-SFG.R | | -0.06 | 0.80 | 0.97 | -0.34 | 0.14 | 0.76 |
| ANG.L-SOG.R | | 0.19 | 0.41 | 0.96 | -0.14 | 0.55 | 0.97 |
| PCUN.L-SPG.R | | 0.08 | 0.73 | 0.97 | -0.70 | 0.001 | 0.04† |
| PCUN.L-CBL.Crus II. R | | -0.19 | 0.39 | 0.96 | -0.07 | 0.76 | 0.97 |
| HES.L-SPG.L | | 0.01 | 0.96 | 0.98 | -0.39 | 0.09 | 0.76 |
| HES.L-CBL.Crus II. R | | 0.18 | 0.44 | 0.96 | -0.10 | 0.68 | 0.97 |
| STG.L-CBL.Crus II. R | | 0.19 | 0.40 | 0.96 | 0.04 | 0.88 | 0.98 |
| CBL.Vermis VI-SFG.R | | 0.08 | 0.72 | 0.97 | -0.18 | 0.45 | 0.96 |
| CBL.Vermis VI-MFG.L | | -0.09 | 0.69 | 0.97 | -0.36 | 0.11 | 0.76 |
| CBL.Vermis VI-MOG.L | | 0.05 | 0.82 | 0.97 | -0.05 | 0.84 | 0.97 |
| CBL.Vermis VI-CBL.Crus I. L | | -0.23 | 0.31 | 0.92 | -0.18 | 0.44 | 0.96 |
| CBL.Vermis VI-CBL.Crus II. R | | 0.01 | 0.96 | 0.98 | -0.40 | 0.08 | 0.76 |
| CBL.Crus I.L-SFG.R | | -0.08 | 0.73 | 0.97 | 0.10 | 0.68 | 0.97 |
| CBL.Crus I.L-MFG.L | | 0.04 | 0.85 | 0.98 | -0.29 | 0.22 | 0.91 |
| CBL.Crus I. L-CBL.Crus II. L | | 0.05 | 0.81 | 0.97 | -0.14 | 0.54 | 0.97 |
| CBL.Crus I. L-CBL.Crus II. R | | -0.01 | 0.97 | 0.98 | -0.01 | 0.98 | 0.98 |
| CBL.Crus I. L-CBL. IX. R | | 0.33 | 0.13 | 0.76 | 0.12 | 0.63 | 0.97 |
| CBL.Crus I.R-SOG.L | | 0.27 | 0.23 | 0.91 | -0.01 | 0.96 | 0.98 |
| CBL.Crus I. R-CBL.Crus II. R | | -0.19 | 0.40 | 0.96 | -0.29 | 0.22 | 0.91 |
| CBL. VIII. L-SPG.L | | 0.24 | 0.28 | 0.92 | -0.25 | 0.29 | 0.92 |
| CBL. VIII. L-SOG.L | | 0.09 | 0.69 | 0.97 | -0.48^*^ | 0.03 | 0.76 |
| CBL. VIII. L-SOG.R | | 0.35 | 0.11 | 0.76 | -0.12 | 0.62 | 0.97 |
| CBL. VIII. L-CBL.Crus II. R | | 0.25 | 0.26 | 0.92 | 0.07 | 0.76 | 0.97 |

SFG.R= right superior frontal gyrus; MFG.L= left middle frontal gyrus; SPG.L= left superior parietal gyrus;SPG.R= right superior parietal gyrus; SMG.R=right supramarginal gyrus; ANG.L= left angular gyrus; PCUN.L=left precuneus; HES.L=left Heschl's gyrus; STG.L=left superior temporal gyrus; SOG.L= left superior occipital gyrus; SOG.R=right superior occipital gyrus; MOG.L= left middle occipital gyrus; DCG.L= left median cingulate gyrus; CBL.Vermis VI=cerebellar vermis VI; CBL.Crus I.L=left cerebellar crus I; CBL.Crus I.R=right cerebellar crus I; CBL.Crus II.L=left cerebellar crus II; CBL.Crus II.R=right cerebellar crus II; CBL.VIII.L=left cerebellar lobule VIII.

ADOS.Social= ADOS Social: ADOS social communication domain affect score; RBS-R total: repetitive behaviors scale-revised total score

Statistical significance *after* FDR correction, †p < 0.05, ∗p < 0.01, ∗∗p < 0.005, ∗∗∗p < 0.001
